# Jejunal gene expression patterns correlate with severity of systemic infection in chicken

**DOI:** 10.1186/1753-6561-5-S4-S4

**Published:** 2011-06-03

**Authors:** Dirkjan Schokker, Mari A Smits, Johanna MJ Rebel

**Affiliations:** 1 Wageningen UR Livestock Research, Animal Breeding and Genomics Centre, Lelystad, P.O. Box 65, 8200 AB, The Netherlands; 2 Central Veterinary Institute, Wageningen UR, Lelystad, P.O. Box 65, 8200 AB, The Netherlands

## Abstract

**Background:**

Not much is known about the effect of *Salmonella enteritidis* on changes in the developmental processes occurring in the intestine of young chicken. Therefore we investigated the correlation of intestinal gene expression patterns with the severity of systemic Salmonella infections.

**Methods:**

The number of Salmonella colony forming units (CFUs) in the liver of infected chicken were plotted against the average intestinal expression profiles of previously identified gene expression clusters. The functional properties of all the genes taken together present in 3 clusters exhibiting positive correlation at early time-points were compared with the functional properties of the genes displaying antagonistic correlations in 1 cluster. The top 5 ranking functional groups were analysed in further detail.

**Results:**

Three clusters showed gene expression profiles which were positively correlated with the severity of systemic disease as measured by the number of Salmonella colony forming units in the liver. In these clusters, genes involved in morphological processes were predominantly present. One cluster had a profile that was negatively correlated with the severity of systemic disease, as measured by numbers of CFUs in the liver. The genes in the latter cluster were mostly involved in cell turn-over and metabolism.

**Conclusions:**

In the developing jejunum of young chicken, both stimulatory and inhibitory gene expression mechanisms are correlated with the severity of systemic Salmonella infections.

## Background

Intestinal development is hallmarked by functional, morphological and immunological development. Genes involved in these three categories have different spatial-temporal expression patterns, as observed in earlier studies [1-4]. Developmental studies are mostly performed in healthy chickens and not much is known of the effect of a disturbance on intestinal development. In a previous [5] study we disturbed intestinal development by oral infection with Salmonella and studied the effects of Salmonella on the immunological development of the intestine for 8 hours post infection (pi) until 21 days pi. Besides the immunological pathways we identified clusters of genes whose expression was correlated with the severity of systemic infection (numbers of CFUs in the liver). In this study, we further analyzed this correlation and focused on the functional properties of genes displaying the correlated expression profiles only in the earlier time points until 4 days pi. This study contrasts other studies that use Salmonella infected chicken to study (innate) immune responses or differences in susceptibilities [6-8].

## Methods

### Design

We used the dataset E-MEXP-2042 from ArrayExpress [9,10], describing whole genome transcriptional profiling of chicken jejunum in a time series (8 hours until 21days pi) after orally infection with Salmonella. The data from Schokker *et al.*[5] was used to define average expression profiles of 9 clusters of genes and to identify the 4 clusters that showed expression profiles correlated with Salmonella counts in the liver. Here we investigate the differences between the positive and negative correlation to the trait in more depth in the first 4 days pi. We combined the genes of the clusters G, H and I (2169 probes), which were found to possess a positive correlation early in time to severity of systemic disease and compared this group of genes to the genes present in cluster F (791) which showed an opposite correlation pattern to the severity of systemic disease. The Database for Annotation, Visualization and Integrated Discovery (DAVID) 6.7 [11] was used for functional annotation clustering of these two sets of genes (March, 2010) [12]. The inputs were lists with chicken gene symbols or the homologous human symbols. Human has a better annotation, 29% of the probes mapped back to a human gene name compared to 24% to a chicken gene name. Also more databases are available for human and the data is analyzed with a human background. The settings in DAVID were similar to the settings as described by Schokker *et al.*[13].

## Results and discussion

**Figure 1:**
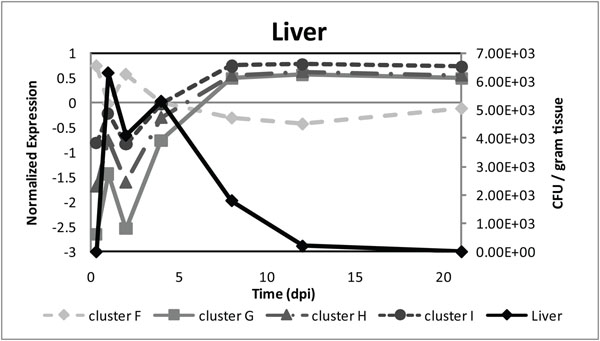
**Correlation of average gene expression patterns of genes present in clusters F–I with Salmonella CFU per gram liver tissue in time.** Cluster F shows negative correlation with the number of Colony Forming Units (CFUs) in liver from 0.33 – 4 days post infection (dpi), whereas clusters G, H and I have positive correlation in that time period.

The average gene expression profiles of the genes in clusters F, G, H, and I showed irregularities at 2 days pi compared to the smooth profiles of the corresponding clusters obtained form non-infected chicken. Clusters G, H, and I showed a positive correlation with the amount of Salmonella colony forming units (CFU) in liver, whereas cluster F showed an anti-correlation (Fig. 1). These positive and negative correlations are based on the period between 8 hours pi to 4 days pi. This correlation suggests that the irregularity in gene expression patterns is most probably directly related to the systemic invasion of Salmonella from the intestinal tract.

The correlations further suggest that these associated genes encode for functional properties related to the severity of systemic disease. To investigate the functional properties, clusters G, H and I were grouped, because of their similar expression pattern and similar association to the number of CFUs in the liver. Subsequently, the genes residing in positively or negatively correlated clusters were used as input for the functional clustering analysis by DAVID. The resulting top 5 of the functional annotation clustering is depicted in Table 1.

**Table 1:** DAVID Functional Annotation Clustering Top 5

Correlation	Rank	Functional Group	ES	Count
Negative	1	Thrombospondins	2.564950833	6
	2	Cellular homeostasis	2.078669595	16
	3	Regulation of activity / Metabolic process	1.969787156	14
	4	Programmed cell death	1.894708614	20
	5	Ion homeostasis	1.825373132	10
Positive	1	Calmodulin (IQ domain)	3.840704976	12
	2	Fibronectin	3.688368085	18
	3	Contractile fiber	3.664863955	15
	4	Cell morphogenesis (neuron)	3.419394841	22
	5	Immunoglobulin I-set	3.397685801	17

Abbreviations: ES, Enrichment Scores

Most functional groups in the positively correlated expression profiles are related to morphological processes, like ‘fibronectin’, ‘contractile fiber’ and ‘cell morphogenesis’. Another top 5 group is ‘Calmodulin (IQ-domain)’, which is involved in multiple processes, like metabolism, inflammation and intracellular movement. Thus this positively correlated group is characterized by major processes involved in both morphological and immunological functions. Apparently, due to transmigration of Salmonella many genes involved in morphological related processes are regulated. Increased transmigration from the gut correlates with increased expression of a number of genes involved in morphological processes in the jejunum, whereas decreased transmigration of Salmonella leads to lower expression of such genes.

Also a specific immune related process ‘immunoglobulin I-set’ is present among the top 5 list. However, the genes contained in this domain are mainly involved in cell adhesion processes. For example vascular (VCAM), intercellular (ICAM), neural (NCAM) and mucosal addressin (MADCAM) cell adhesion molecules, as well as junction adhesion molecules (JAM) [14]. Some of these genes are also involved in immune cell adhesion, for example ICAM1 and VCAM1 are involved in monocyte-endothelial adhesion [15]. Moreover JAM genes are known to be involved in lymphocyte homing [16]. The expression of these adhesion genes is directly and positively related to Salmonella transmigration and the severity of systemic disease.

In the negatively correlated group high expression is observed at 8 hours pi, followed by stable expression around zero until 21 days pi. Compared to the corresponding cluster of non-infected chicken, in the Salmonella disturbed chicken this cluster showed an irregularity in gene expression profile opposite to the Salmonella load in liver. This peak in gene expression may reflect a feedback mechanism of the jejunum. In this cluster, functional groups like ‘thrombospondin’, homeostasis related processes and ‘programmed cell death’ are observed, which are mainly involved in cell turn-over processes. The thrombospondin family is related to adhesive glycoproteins, and is involved in various processes like adhesion/migration, cytoskeletal organization, proliferation, phagocytosis, apoptosis and platelet aggregations [17,18]. The functional annotation clustering results furthermore indicate that in this cluster, genes are also involved in programmed cell death. After infection with Salmonella at day zero different processes are initiated and also the Salmonella load increases in liver (Fig. 1). Many cells will die or loose functionality due to the infection, therefore it necessary for the host to respond by replacing infected and affected cells. Thus the majority of genes residing in the negatively correlated group are associated to cell turn-over processes suggesting that due to the transmigration of Salmonella across the intestinal mucosa, processes for tissue repair are induced almost immediately. However the data suggest that the genes involved in such processes have a delayed response to the Salmonella infection, therefore a peak in gene expression is observed after the peak of Salmonella load in the liver.

In addition to the turn-over processes, also processes involved in ‘homeostasis’, maintenance of an internal steady-state at the level of the cell are observed. These latter processes can be characterized as metabolic.

## Conclusions

Severity of systemic Salmonella infections can be associated to gene expression patterns in the jejunum. Negatively correlated gene expression patterns correspond to processes involved in metabolism, cell turn-over and tissue repair. Positively correlated gene expression patterns are associated with morphological and immunological related processes.

## Competing interests

The authors declare that they have no competing interests.
